# Methods of Intracanal Reinforcement in Primary Anterior Teeth–Assessing the Outcomes through a Systematic Literature Review

**DOI:** 10.5005/jp-journals-10005-1282

**Published:** 2015-04-28

**Authors:** Neeti Mittal, Hind Pal Bhatia, Khushtar Haider

**Affiliations:** Assistant Professor, Department of Pedodontics and Preventive Dentistry Santosh Dental College, Ghaziabad, Uttar Pradesh, India; Professor and Head, Department of Pedodontics and Preventive Dentistry, Manav Rachna Dental College, Faridabad, Haryana, India; Postgraduate Student, Department of Pedodontics and Preventive Dentistry Santosh Dental College, Ghaziabad, Uttar Pradesh, India

**Keywords:** Evidence, Intracanal reinforcement, Posts, Primary teeth, Restorations.

## Abstract

**Aim:** To assess how the various methods of intracanal reinforcement (short root canal posts) performed in their clinical and radiographic outcomes for restoring grossly broken down primary anterior teeth after pulpectomy for 1 year or longer follow-up period.

**Materials and methods:** Literature search of electronic databases (Sept 2013) and various journals (1980-Sept 2013) using medical subject headings and free text terms was conducted. For inclusion in quality assessment, prespecified inclusion criteria were applied. Quality assessment was performed by using ‘The Cochrane collaboration’s tool for assessing risk of bias’.

**Results:** Seven relevant papers were selected for full text evaluation. After applying the inclusion criteria, only two trials could be considered for quality assessment. Both of these were classified as having high risk of bias.

**Conclusion:** The evidence to support any method of intracanal reinforcement for restoring grossly broken down anterior teeth is presently lacking. Further trials with well-defined methodology are needed.

**How to cite this article:** Mittal N, Bhatia HP, Haider K. Methods of Intracanal Reinforcement in Primary Anterior Teeth– Assessing the Outcomes through a Systematic Literature Review. Int J Clin Pediatr Dent 2015;8(1):48-54.

## INTRODUCTION

The phenomenal developments in the field of preventive dentistry have worked wonders to cause a remarkable decline in prevalence of dental caries globally.^1,2^ But, yet, there remains a section of young population from developed as well as developing countries who present as high caries risk subjects.^3^ It is not very uncommon to see young patients with multiple grossly decayed anterior as well as posterior primary teeth. Restoring primary teeth is important not only for mastication, speech, alveolar growth and harmonious stomatomusculoskeletal system, but also for psychological well-being of the child. Restoring grossly carious primary teeth is challenging compared to permanent teeth which have greater bulk of tooth structure to offer promising retention for restorations. Pulpal involvement in primary teeth is faster and endodontic intervention further leaves very little tooth structure. It is very common to see primary anterior teeth with complete coronal destruction. To prepare these mutilated primary anterior teeth to receive complete coronal restorations, retention is gained from short intracanal posts.

Gaining intracanal retention for restoring mutilated anterior teeth is tricky compared to permanent teeth not only because of little remaining tooth structure; but also because of the fact that primary teeth have to make way for their permanent counterparts. The intracanal posts should shed in a timely manner to allow unimpeded timely eruption of their permanent successors in normal undefected positions. Other much needed characteristics of intracanal posts for primary teeth are biocompatibility, ease of availability and applicability, esthetics and ability to withstand masticatory forces.

A multitude of methods have been used for intracanal reinforcement for anterior teeth, such as short composite posts,^4,5^ short wire posts (omega loop),^6-10^ Ni-Cr coils pring posts,^11^ ready made glass fiber posts,^9,12-15^ polyethylene fibre post/ribbond^16-21^ and metal screw posts.^22,23^ The crown anatomy can be restored by direct composite build up by incremental method,^6,8,16,20,22^ composite build-up using celluloid strip crowns,^4,7,14,18,19,23^ composite build-up by indirect technique^11,12,17,24^ and biological shell crowns.^8,25^

Owing to heterogeneity of available data on intracanal posts, the pediatric dentists might encounter difficulty in having an evidence-based choice of intracanal posts in clinical situations. Keeping this in mind, it was decided to perform a systematic review to assess the quality of evidence for methods of intracanal reinforcement for grossly broken down primary anterior teeth. Our primary study objective was to assess how the various methods of intracanal reinforcement (short root canal posts) performed (in their clinical and radiographic outcomes) for restoring grossly broken down primary anterior teeth after pulpectomy in a follow-up period of 1 year or more.

**Table 1:** **Table 1:** Literature search strategy and outcomes

*Search method*		*Search term/journal name*		*Number of items retrieved*		*Number of items related to study outcome*		*Items not found during PubMed search*		*Case reports*		*In vitro studies*		*Number of relevant items (in vivo clinical trials)*	
PubMed search		Intracanal post and primary not permanent teeth		10		6		NA		2		2		2	
PubMed search		Canal and post and primary not permanent teeth		61		11		NA		6		3		2	
PubMed search		Restoration and anterior teeth and primary not permanent teeth		69		10		NA		6		2		2	
Scopus		Restoration and anterior teeth and primary teeth		154		12		2		6		1		5	
Cochrane library		Restoration and anterior teeth and primary not permanent teeth		2		0		0		0		0		0	
Hand search		International Journal of Pediatric Dentistry		NA		1		0		1		0		0	
Hand search		Journal of Clinical Pediatric Dentistry		NA		9		1		3		3		3	
Hand search		Journal of Dentistry for Children		NA		1		1		1		0		0	
Hand search		Pediatric Dentistry		NA		0		0		0		0		0	
Hand search		Journal of American Dental Association		NA		1		1		0		0		1	
Total				–		–		–		15		5		7	

## MATERIALS AND METHODS

*Search strategy*: The literature studying the ‘methods of intracanal reinforcement’ for restoring grossly decayed primary anterior teeth was reviewed by Neeti Mittal (NM) and Khushtar Haider (KH) independently and in duplication. In addition to electronic databases, hand search was also performed for some journals (Table 1). Controlled vocabulary using MeSH terms and combination of free text terms was used (Table 1). Following databases were searched: PubMed (till Sept 2013), Scopus (till Sept 2013) and Cochrane library (till Sept 2013). Search strategy was developed for PubMed and was modified appropriately for other databases. There were no language restrictions and no filters were activated. Titles and abstracts were assessed by NM a nd KH independently for inclusion in this review. In case of any doubt, consensus was arrived at by seeking opinion of Hind Pal Bhatia (HB).

*Selection criteria*: For full text evaluation, references were selected by reviewing titles and abstracts by NM and KH independently and in duplication. Descriptive data extraction was performed for all identified prospective clinical trials studying the clinical and radiog raphic outcomes of intracanal reinforcement for restoring grossly broken down primary anterior teeth after pulpectomy. After descriptive data extraction, inclusion criteria for consideration in qualitative systematic review were applied. These inclusion criteria were: prospective randomized controlled clinical trial, clinical and radiographic performance of intracanal posts for restoring grossly decayed primary anterior teeth as outcome measure, follow-up period of 1 year or more. Case reports and *in vitro* trials were not included.

*Data extraction*: Data were extracted by NM for 7 items, i.e. author, year, sample, groups, evaluation criteria, results and author’s conclusions. In case of missed data, corresponding authors were contacted for gathering necessary information.

*Quality assessment and risk of bias*: Quality assessment was performed only after applying the prespecified inclusion criteria. It was done independently and in duplication by NM and KH. Doubts were resolved by consensus of all three authors.

Quality assessment was done using a modified version of ‘The Cochrane Collaboration’s tool for assessing risk of bias’.^26^ Risk of bias was evaluated for following domains, i.e. selection bias, performance bias, detection bias, attrition bias, reporting bias and miscellaneous. Multiple parameters were used to assess these domains (Table 2). Risk of bias was calculated as high risk, low risk or unclear risk of bias as per recommendations provided in online version of Cochrane handbook for systematic reviews of interventions (version 5.1.0; updated on March 2011). ^26^ Overall risk of bias for individual study was calculated as per prespecified criteria. If all parameters were reported to have low risk of bias, the study was said to have low risk of bias. If one parameter was reported to have high risk of bias, the study was said to have moderate risk of bias. If one parameter was reported to have unclear risk of bias, the study was said to have unclear risk of bias. Further, any study was considered to have high risk of bias if ≥2 parameters had unclear and/or high risk of bias.

## RESULTS

Out of a total of 27 references retrieved, 15 case reports and 5 *in vitro* trials were excluded from full text evaluation. A total of 7 *in vivo* clinical trials were identified for data extraction (Table 3).

*Quality assessment*: Only two trials ^8,9^ were considered for quality assessment as 5/7 c linical trials were excluded (Table 4). Reasons for exclusion are given in Table 4. Out of a total of 12 parameters evaluated, high risk of bias was reported for nine parameters in trial reported by Subramaniam et al.^9^ The same was the case for trial reported by Grewal and Seth,^8^ where a total of six parame ters were found to have high risk of bias (Table 5). Hence, the overall risk of bias assigned to both of these trials was ‘high’.

*Meta-analysis*: Data could not be pooled to perform meta-analysis as only limited number of heterogeneous studies could be included in this systematic review. Further, the evidence generated from these two studies was also poor as both were classified as having high risk of bias.

**Table 2:** **Table 2:** Assessing the risk of bias: domains and parameters

*Domain*		*Parameters*	
Selection bias		1. Definition of inclusion criteria	
		2. Definition of exclusion criteria	
		3. Random sequence generation	
		4. Allocation concealment	
Performance bias		Adequate blinding	
Detection bias		Blinding of outcome assessor	
Attrition bias		Reporting of dropouts	
Reporting bias		Incomplete outcome reporting	
Miscellaneous		1. Elaboration of clinical assessment methods and parameters	
		2. Elaboration of radiographic assessment methods and parameters	
		3. Adequate follow-up period	

## DISCUSSION

The evidence to base any recommendation could not be gathered in present systematic review owing to small number (only 2) of poor quality randomized trials.^8,9^

In order to generate quality evidence, the trials should have adequate inclusion and exclusion criteria, i.e. adequate definition of clinical and radiographic conditions, e.g. amount of remaining tooth structure (as this is important to support post and subsequent crown) and/ or mobility and/or remaining root length. In addition to this, another very important factor to ensure baseline equivalence, randomization should be ensured via random sequence generation and allocation conceal-ment.^27-30^ None of these measures were incorporated in selected trials and, thus, these studies were flawed with selection bias. Blinding is important to avoid detection bias inatrial. ^27,29,30^ In both of the selected studies, no statements were made regarding blinding. Blinding of participants should be done; however, blinding of operator, in such trials is not feasible owing to clear distinction between different types of posts as well as obvious differences in clinical techniques. Instead, blinding of outcome assessor should be done.^29,30^ In addition to type of posts and/or techniques, the outcome assessor should also be additionally blinded to time period of evaluation, e.g. immediate follow-up or 6 months, 12 months and so on. Another methodological flaw with selected studies was lack of information about drop outs, if any. Drop outs in any clinical study can disrupt the baseline equivalence amongst study groups.^26^ Postoperative evaluation at multiple time intervals should be done using prespeci-fied clinical and radiographic criteria. These criteria should be able to evaluate the success of material as well as technique. Both of these studies used prespeci-fied criteria, but criteria used by Subramaniam et al^9^ did not include any radiographic measures. Also, the clinical criteria used by them were neither sufficient nor validated. Ideally, such trials should have a follow-up period to allow observation of normal exfoliation and eruption of permanent successors. But, this is always not feasible. A follow-up period of sufficiently long duration to reasonably prove the successful performance in oral cavity for predefined clinical and radiographic criteria can serve as a proxy for this. In this systematic review, a follow-up period of 1 year was considered to be sufficient.

A reliable way to ensure quality in clinical randomized trials to generate quality evidence is to follow, ‘The Cochrane Collaboration’s tool for assessing risk of bias’^26^ and ‘CONSORT statement’.^31^

As the evidence to support any of the material and/ or technique was found to be deficient, a narrative discussion is provided below to guide pediatric dentists to select a suitable method of intracanal reinforcement in commonly encountered clinical problems.

*Short composite posts*: Short composite posts are easy to apply and exhibit excellent esthetics because of translucency of composite resin. On the contrary, wire/ metal posts may exhibit greyish translucency due to color of wire not being masked completely by overlying resin. Another advantage is easy technique. However, with composite resin posts there is always an inherent risk of loss of retention owing to polymerization shrinkage.^5,32^

**Table 3:** **Table 3:** Data extraction from *in vivo* clinical trials

*Author/year*		*Sample*		*Groups*		*Evaluation criteria*		*Results*		*Author’s conclusion*	
Judd PL et al 1990^5^		N = 92 teeth		Short composite post with composite resin crown		Marginal integrity, mobility, caries at the composite resin–tooth margin and fractures at 6 and 12 months		Four teeth in two patients showed recurrent caries at the composite resin-tooth cervical margin. Three of these teeth were restored and one was extracted. Three crowns showed incisal fracture of minimal severity. These were later rebuilt with a resin add on technique. Four crowns displayed severe attrition in one patient who was a severe bruxer.		Short posts were retentive. Recurrent caries and severe bruxism–factors beyond operator control–posed some problems that were readily resolved.	
Sharaf AA 2002^15^		N = 12 Age = 4 years		N = 30 teeth Fiber glass post with celluloid strip crown		Color match, marginal adaptation, marginal discoloration, anatomic form, secondary caries, gingival condition, pain, temperature sensitivity and periapical condition at 3, 6, 9 and 12 months		28/30 teeth performed well. Failure in pulp treatment rather than failure of the restoration itself was reported in 2/30 teeth.		This technique significantly improved the fracture load resistance of composite celluloid crown.	
Mortada A, King NM 2004^6^		N = 25 Age = 38 months		N = 96 teeth Omega-shaped wire post with compomer		Retention, recurrent caries and the presence of any periapical radiolucency at 3, 6, 12 and 18 months		In two patients although the restorations were intact, the endodontic procedure was considered to have failed. By the 18-month recall, 81.2% teeth were available for examination and of these there was complete retention of the restorations on 79.9% of the teeth.		The technique for restoring primary anterior teeth was simple, quick and effective.	
Grewal N, Seth R 2008^8^		N = 32 Age = 3-5 years		Group 1 (n = 75): Biologic post and crown Group 2 (n = 75): short composite post		Modified USPHS system applied every 0, 3, 6, 9 and 12 months		Clinical performance of biological post and crown restorations and intracanal reinforced composite restorations was comparable with respect to shade match, marginal discoloration, marginal integrity, surface finish, gingival health, retention, and recurrent carious lesions.		The biological restoration presented as a cost-effective, clinician-friendly, less-technique sensitive and esthetic alternative to commercially available restorative materials used for restoring grossly carious deciduous teeth.	
Subramaniam P et al 2008^9^		N = 10 Age = 3-4 years		Group 1 (n = 14): Fiber glass post with celluloid strip crowns Group 2 (n = 14): Omega-wire post with celluloid strip crowns		Retention and marginal adaptation at 1, 6 and 12 months		Fiber glass posts showed better retention and marginal adaptation than omega-shaped stainless steel wire posts.		Glass fiber posts show better retention and marginal adaptation than omega-shaped stainless steel wire posts.	
Aminabadi NA, Farahani RM 2009^10^		N = 60 Age = 3-4 years		N = 144 teeth Omega-shaped wire post with compomer		Retention, recurrent caries and the presence of any periapical radiolucency at 6, 12 and 24 months		The failure rates after 12 and 24 months were 10.8% and 18.5% respectively. The primary canines exhibited minimum loss of the restorative material. Two teeth exhibited pathological mobility after 2 years. There were not any signs of root fracture or recurrent caries in any of the restored teeth.		The modified omega loop is an efficient technique. The ease of manipulation and short chairside time are further advantages of the technique.	
Memarpour M, Shafei F 2013^21^		N = 24 Mean age = 4.2 years		N = 55 teeth Polyethylene ribbon fibers followed by composite resin		Modified Ryge criteria every 6 months for 30 months		The surface textures for most of the restorations were judged as excellent. There was no evidence of significant changes in marginal integrity. Most restored incisors (81%) received an Alpha rating for retention. The baseline and recall retention scores differed significantly (p = 0.002).		Polyethylene fiber posts along with extensive composite restorations showed excellent clinical performance.	

*Polyethylene ribbond fiber posts*: Ribbond fiber posts offer good impact strength to composite resin used for coronal reconstruction. This is because of their modulus of elasticity and flexural strength being close to dentine.^17,18,33^ Another advantage offered is better adhesion to composite resin matrix when compared to glass fiber posts.^34^ The excellent translucency offers satisfactory esthetics. Best reason for selection of ribbond fiber posts is ease of insertion and when used with flowable composites they conform to shape of root canal.

*Omega wire posts*: Wire extensions bent in different shapes, i.e. alpha,^35^ gamma^36^ and delta,^37^ have long been used by many clinicians as posts for primary teeth. Wire bent in alpha shape is pressure bonded inside the root canals and this may lead to stresses in the dentin. Although with wire bent in gamma shape a success rate of 93% has been reported;^36^ the technique, however, has been rated as being operator dependent. Only disadvantages with this technique is only two point retention obtained and color of wire being visible through overlying resin.

*Biologic posts*: As discussed above, the prime factors to be borne in mind while selecting the appropriate intra-ca nal posts are biocompat ibility, ease of applicability and availability with requirement for lesser chairside time. Dentine post/post with core have all these characteristics and an additional advantage of being inexpensive.

Previously, the dentin posts have been prepared using primary root dentin^8,24,25^ while premolar root dentin can also be used. Latter, being the most common tooth extracted for orthodontic reasons, are widely available, while former have a limited availability. Another advantage of using the premolar root pieces is ease of finding them in sound form, while it is difficult to get primary root dentine free from resorption.

**Table 4:** **Table 4:** Studies excluded from systematic analysis

*Author and year*		*Reason for exclusion*	
Judd et al (1990^5^)		Absence of control group	
Sharaf (2002^15^)		Absence of control group	
Mortada and King (2004^6^)		Absence of control group	
Aminabadi and Farahani (2009^10^)		Absence of control group	
Memarpour and Shafei (2013^21^)		Absence of control group	

One of the limitations of using biologic restorations is preoperative preparation, such as sterilization and preparation of natural tooth to make dentine post/post and core/shell crown. But, these steps can be performed by dental auxiliaries also, and the dentist does not need to spare time for this.

Some parents may find this technique objectionable and inacceptable. However, after counseling and assurance by pediatric dentist about harmless nature of this restorative modality, this problem can be resolved easily.

**Table 5:**
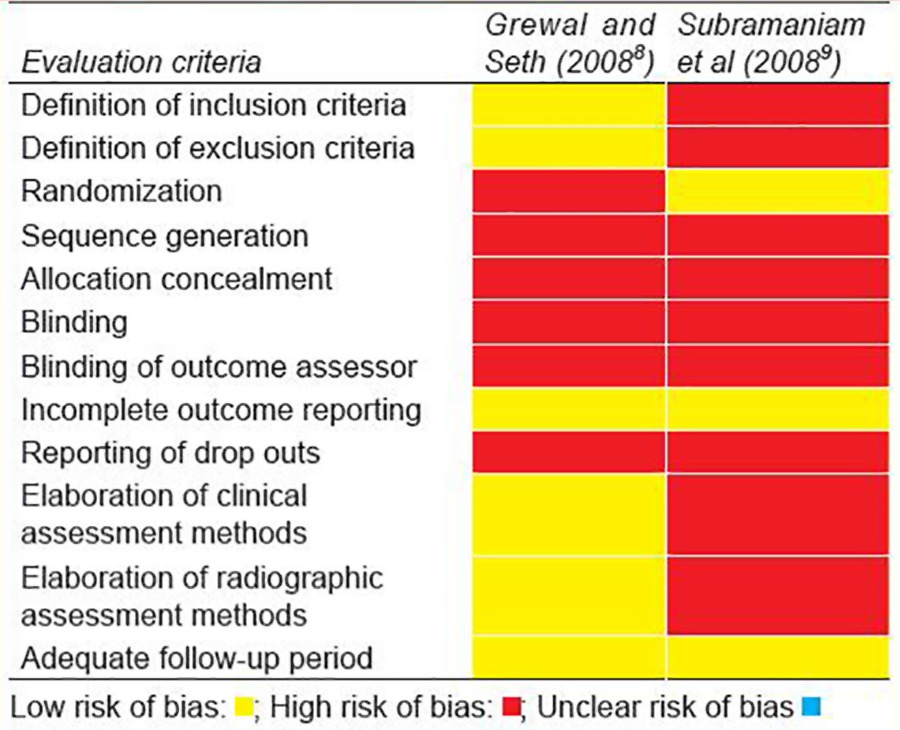
**Table 5:** Quality assessment of included studies

*Indirect composite resin posts*: Previously, few authors have reported restoring grossly broken down anterior teeth by indirect technique using various types of posts, such as preformed Ni-Cr posts,^11^ fiber glass posts^12-14^ and ribbond^17^ as intracanal reinforcement. All of the above listed methods require longer chairside time which may compromise the cooperation by young child with short attention span and little patience. Instead of this usual two step technique, composite crown and post can be fabricated as a single unit by indirect method, thus, saving the chairside time.

Direct composite restorations have been associated with marginal microleakage following polymerization shrinkage, especially at the cervical cavosurface margins,^38^ improper contact points^39^ and relatively low wear resistance.^40^ Extraoral improved curing of the composite resin^39^ can minimize above-mentioned disadvantages of direct composite restorations.

Specific systems though available for laboratory processed indirect composites have little use for primary teeth. The commercially available indirect systems have greater filler loading for improved mechanical strength and better handling properties, these pose a greater economic burden owing to higher cost. Further, these systems were developed for permanent teeth which have a much longer time period to serve in oral cavity than primary teeth. The direct composite material being routinely used for direct restorative procedures may also be used and this may be cured with same light cure unit being routinely used for direct composite restorations. Apart from economic advantage, another benefit with direct composite material is that it promises to wear at a rate synonymous with primary teeth.

From above discussion, it becomes clear that further trials with well-defined methodology to eliminate any bias are needed. Currently, the evidence is lacking to provide any recommendation about any method of intracanal reinforcement. The criteria to select method of intracanal reinforcement, i.e. type of posts to restore grossly mutilated teeth are biocompatibility, ease of availability and applicability, esthetics, ability to withstand masticatory forces and ability to allow uninterrupted eruption of permanent successors.

The choice of type of post and/or technique should be based on clinical condition of tooth to be restored, finances and operator as well as patient and/or parent’s preference.

## CONCLUSION

1. The evidence to support any method of intracanal reinforcement for restoring grossly broken down anterior teeth is presently lacking.
2. Further, trials with well-defined methodology should be conducted keeping in mind ‘The Cochrane Collaboration’s tool for assessing risk of bias’ and ‘CONSORT statement’.
3. The choice of type of post and/or technique is based on clinical condition of tooth to be restored, finances and operator as well as patient and/or parent’s preference.
